# Changes in clot lysis levels of reteplase and streptokinase following continuous wave ultrasound exposure, at ultrasound intensities following attenuation from the skull bone

**DOI:** 10.1186/1471-2261-8-19

**Published:** 2008-08-26

**Authors:** Bjarne Madsen Härdig, Jonas Carlson, Anders Roijer

**Affiliations:** 1 Department of Clinical Sciences, Cardiology, Lund University, Lund University Hospital, Lund, S221 85, Sweden

## Abstract

**Background:**

Ultrasound (US) has been used to enhance thrombolytic therapy in the treatment of stroke. Considerable attenuation of US intensity is however noted if US is applied over the temporal bone. The aim of this study was therefore to explore possible changes in the effect of thrombolytic drugs during low-intensity, high-frequency continuous-wave ultrasound (CW-US) exposure.

**Methods:**

Clots were made from fresh venous blood drawn from healthy volunteers. Each clot was made from 1.4 ml blood and left to coagulate for 1 hour in a plastic test-tube. The thrombolytic drugs used were, 3600 IU streptokinase (SK) or 0.25 U reteplase (r-PA), which were mixed in 160 ml 0.9% NaCl solution. Continuous-wave US exposure was applied at a frequency of 1 MHz and intensities ranging from 0.0125 to 1.2 W/cm^2^. For each thrombolytic drug (n = 2, SK and r-PA) and each intensity (n = 9) interventional clots (US-exposed, n = 6) were submerged in thrombolytic solution and exposed to CW-US while control clots (also submerged in thrombolytic solution, n = 6) were left unexposed to US.

To evaluate the effect on clot lysis, the haemoglobin (Hb) released from each clot was measured every 20 min for 1 hour (20, 40 and 60 min). The Hb content (mg) released was estimated by spectrophotometry at 540 nm. The difference in effect on clot lysis was expressed as the difference in the amount of Hb released between pairs of US-exposed clots and control clots. Statistical analysis was performed using Wilcoxon's signed rank test.

**Results:**

Continuous-wave ultrasound significantly decreased the effects of SK at intensities of 0.9 and 1.2 W/cm^2 ^at all times (P < 0.05). Continuous-wave ultrasound significantly increased the effects of r-PA on clot lysis following 20 min exposure at 0.9 W/cm^2 ^and at 1.2 W/cm^2^, following 40 min exposure at 0.3, 0.6, 0.9 and at 1.2 W/cm^2^, and following 60 min of exposure at 0.05 0.3, 0.6, 0.9 and at 1.2 W/cm^2 ^(all P < 0.05).

**Conclusion:**

Increasing intensities of CW-US exposure resulted in increased clot lysis of r-PA-treated blood clots, but decreased clot lysis of SK-treated clots.

## Background

Ultrasound (US) has been used to enhance thrombolytic therapy, for example, in the treatment of stroke. In this setting, US is usually applied over the temporal bone, exposing the obstructed vessel to US concomitantly with treatment with thrombolytic drugs [1-3]. The enhancement of various thrombolytic drugs has been demonstrated during *in vitro *clot lysis at frequencies, ranging from 20 kHz to 4.5 MHz [4-7]. Positive effects on clinical outcome have been reported when high frequency US has been used *in vivo *[1,8]. However, in the CLOTBUST trial, the effects were found not to be statistically significant [1]. This is in contrast to the results from *in vitro *studies of US-enhanced thrombolysis, where considerable enhancement effects of the clot lysis have been shown as a result of exposure to US [7,9,10]. This discrepancy might be explained by the attenuation of US intensity passing through the temporal bone structure during high frequency US exposure, although direct comparison between *in vitro *and *in vivo *results should be made carefully one possible explanation for the different levels seen could be attenuation induced by the skull bone. There have been reports of decreases in the output intensity between 86.8% and 99.2% when US is applied over the temporal bone [11,12]. Low frequency US on the other hand, has greater penetration through bone tissue compared to high frequency US, which results in higher US intensities reaching the obstructed vessel [13,14]. However, low frequency US has been shown to induce a higher rate of bleeding complications during US-enhanced thrombolysis *in vivo *[15].

Other factors of US than intensity and frequency also seem to affect the results during US-enhanced thrombolysis. We have previously only found, during pulsed-wave US SK induced clot lysis, enhanced effects at low intensity (0.5 W/cm^2^) [16,17]. During pulsed-wave US exposure of r-PA induced clot lysis, enhancement effects occur both at high and low intensities (i.e. ≤ 0.25 W/cm^2 ^or > 2.0 W/cm^2^) [18]. The enhancement effects might thus depend on duty cycle, i.e. the number of pulses sent [9,19,20]. Meunier et al reported increasing effects on tissue type plasminogen activators mediated clot lysis depending on increasing duty cycle [20]. However, Holland et al failed to verify the same duty cycle dependency [9]. Others have shown higher grades of enhancement using CW-US exposure than when pulsed-wave US exposure was used [5,21].

The aim of this study was to investigate the changes in the effect of clot lysis of r-PA and SK during low-intensity, high-frequency CW-US exposure, intensities within the area following attenuation from the skull bone.

## Methods

The methods employed for clot formation and clot lysis evaluation, and the ultrasonic properties of the model have been described in detail previously [17,18,22]. Only a brief description will thus be given below.

### Clot formation

Blood clots were made using fresh venous blood from seven healthy volunteers (3 men and 4 women, age 47.5 ± 12.5 year (mean ± SD)) not receiving anticoagulation treatment and with no history of coagulation disturbances. After collection the blood was immediately transferred to a Teflon-coated bottle. The collected blood was then anticoagulated using citrate-phosphate-dextrose adenine (CPDA). Each blood clot was made from 1.4 ml CPDA-anticoagulated blood to which 0.025 mmol CaCl_2 _had been added to induce coagulation. The blood was then left to coagulate around a wool yarn (100 m/54 g, Peer Gynt, Sandnes Uldvarefabrik A/S, 4300 Sandnes, Norway) in a plastic test-tube for one hour [17,18,22].

### Determination of clot lysis

Following one hour of coagulation, the clot was carefully extracted together with the wool yarn and mounted in a plastic frame that was lowered into a clot container with 160 ml of r-PA or SK mixed NaCl solution [17,18,22].

To evaluate clot lysis 1 ml samples of the thrombolytic solution were taken from the clot container every 20 minutes during one hour (20, 40 and 60 min) to estimate the haemoglobin (Hb) leakage from the clot. The sample was added to 4 ml of Drabkins solution and the Hb content (mg) was measured by spectrophotometer at 540 nm, as described elsewhere [23]. To determine clot lysis the loss of Hb (mg) in each individual clot (following 20, 40 and 60 min of exposure) was divided by the Hb content (mg) of a fully lysed clot (from each volunteer), resulting in an estimation of percentage clot lysis (equation 1) [22].

(1) $$\%\text{ clot lysis}=\frac{\text{experimental clot Hb}}{\text{fully lysed clot}}(\times 100)$$

### Thrombolytic drugs

Two thrombolytic drugs were used in the present study, 0.25 U of r-PA (Rapilysin 10 U^®^, Roche Registration Ltd, Hertfordshire, Great Britain) was mixed in 160 ml 0.9% NaCl solution resulting in a concentration of 0.001562 U/ml. The other was SK, (Streptase^®^, 1.5 million international units, Hoechst Marion Roussel AB, Stockholm, Sweden), and 3600 IU mixed in 160 ml 0.9% NaCl solution with a resulting concentration of 22.5 IU/ml. These concentrations of the thrombolytic drugs are optimised for use in this *in vitro *method, the optimisation procedure has been described in detail previously [22].

### Ultrasound exposure

**Figure 1:**
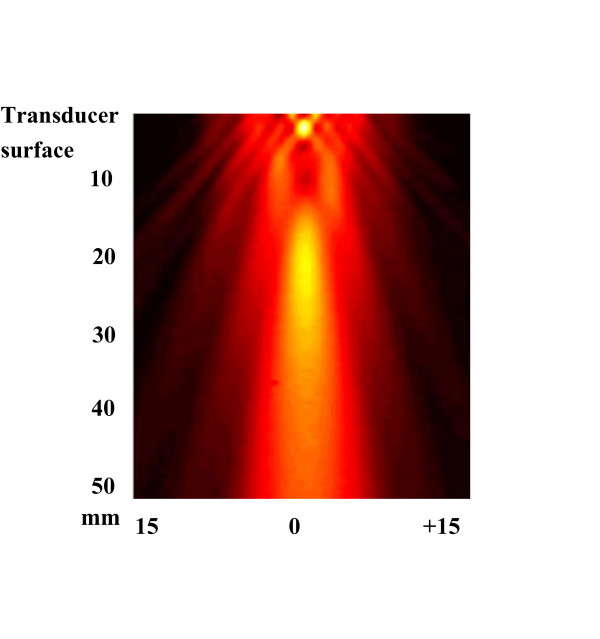
**The field distribution for the transducer used in the present study**. Needle hydrophone exploration of field distribution for the transducer. Scanning was performed over an area of 50 × 30 mm^2 ^in the y- and z-direction starting close to the transducer surface. No exact values of intensity were measured. Clots were placed 30 mm from the transducer surface.

Continuous-wave US emitted by an unfocused piezoelectric transducer (CERAM AB, Lund, Sweden) with a resonance frequency of 1 MHz (diameter = 16 mm, area = 2.0 cm^2 ^and a near field ending 42 mm from transducer surface) was used in all experiments. The transducer was excited by an electronic system consisting of a function generator (HP 3314A, Hewlett-Packard, Washington, USA) and an RF power amplifier (ENI 240L, ENI, Rochester, New York, USA). Prior to experiments the transducer were calibrated by determining spatial-average temporal-average intensity in W/cm^2 ^by measuring the total pressure of US radiation on an electrical balance (Model UPD-DT-1, Ohmic Instrumental co). Needle hydrophone exploration of field distribution for the transducer were performed in degassed water, but exact values of intensity were not measured (Figure 1).

The effect of one hour of 1 MHz CW-US exposure, at intensities 0.0125, 0.025, 0.05, 0.1, 0.15, 0.3, 0.6, 0.9 and 1.2 W/cm^2 ^on clot lysis induced by either SK or r-PA was evaluated. For each thrombolytic drug (n = 2, SK and r-PA) and each intensity (n = 9) interventional clots (US-exposed, n = 6) were submerged in thrombolytic solution and exposed to CW-US while control clots (also submerged in thrombolytic solution, n = 6) were left unexposed to US. Thus, the total number of clots used were 216.

### Statistical analysis

Wilcoxon's signed rank test, was used to assess differences between interventional and control clots at each intensity, following 20, 40 and 60 min of exposure. In all statistical comparisons, P-values below 0.05 were considered significant.

### Ethical considerations

The experiments described in the present study were conducted with the consent of each participant, and were approved by the Regional Ethical Review Board in Lund (approval: 879/2004).

## Results

### Streptokinase treated clots

**Figure 2:**
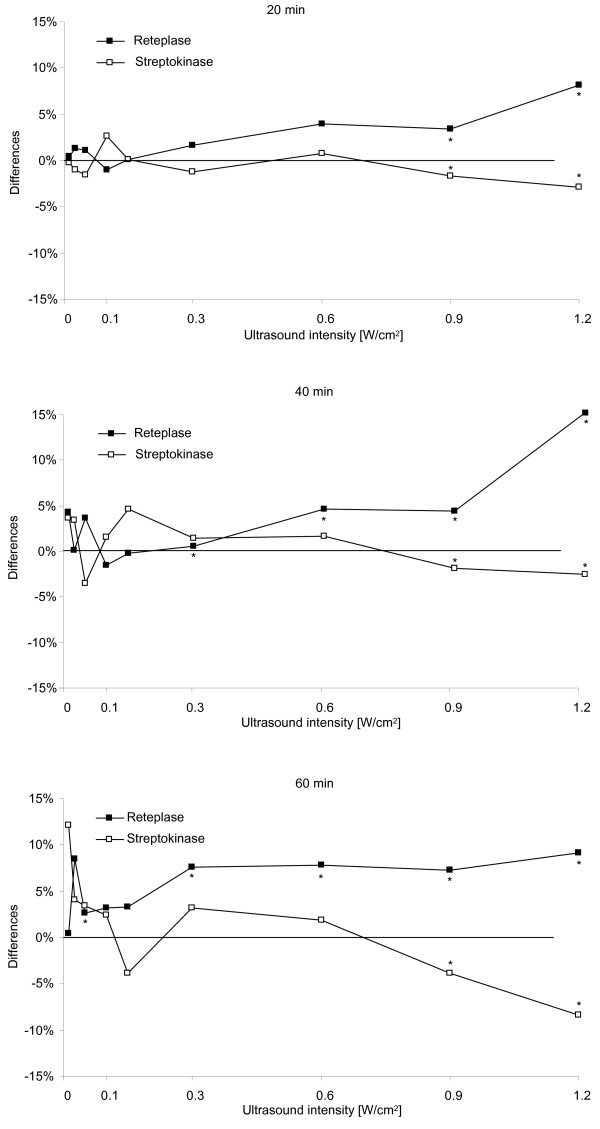
**Difference in clot lysis following continuous-wave ultrasound exposure**. Difference in clot lysis following 20, 40 and 60 min of continuous-wave ultrasound exposure at different intensities, presented as the difference between ultrasound-exposed clots and control clots: □ = clots exposed continuous-wave ultrasound and streptokinase (n = 6), ■ = clots exposed to continuous-wave ultrasound and reteplase (n = 6). Wilcoxon's signed rank test was used to assess statistical differences, * = P < 0.05.

Statistically significant decreases in clot lysis were seen at 0.9 W/cm^2 ^following 20 min (-2%), 40 min (-2%) and 60 min (-4%) CW-US exposure of SK-treated clots (P < 0.05 in all cases). At an intensity of 1.2 W/cm^2 ^the decrease in clot lysis following 20 min of CW-US exposure was 3% and following 40 and 60 min of CW-US exposure 3 and 8%, respectively (P < 0.05 in all cases) (see Table 1 and Figure 2). No increase in clot lysis was seen in clots treated with SK at any time or intensity of CW-US exposure.

**Table 1:** Difference in clot lysis following continuous-wave ultrasound exposure.

**Intensity (W/cm^2^)** **Time (Min)**	**US+SK (n = 6)**	**Control (n = 6)**	**P-value**	**US+r-PA (n = 6)**	**Control (n = 6)**	**P-value**
**0.0125**						
**20**	13 (11–29)	13 (11–29)	0.69	7 (5–11)	6 (4 – 9)	0.08
**40**	27 (19–47)	23 (21–36)	0.25	16 (9–22)	12 (7–25)	0.25
**60**	43 (22–59)	31 (23–54)	0.12	22 (14–42)	22 (10–43)	0.46
**0.025**						
**20**	15 (13–26)	16 (13–31)	0.34	9 (5–12)	7 (3–10)	0.25
**40**	25 (18–33)	22 (19–40)	0.60	16 (10–19)	15 (7–17)	0.17
**60**	34 (21–58)	30 (23–49)	0.25	36 (13–37)	27 (10–37)	0.12
**0.05**						
**20**	15 (12–27)	17 (15–21)	0.69	8 (4–10)	7 (5–10)	0.46
**40**	21 (18–35)	25 (21–36)	0.08	16 (13–19)	12 (11–22)	0.34
**60**	39 (28–52)	36 (27–52)	0.46	26 (20–30)	24 (17–28)	<0.05
**0.1**						
**20**	18 (13–27)	15 (10–31)	0.69	7 (6–10)	8 (4–9)	0.50
**40**	22 (19–37)	21 (15–42)	0.35	15 (10–18)	17 (3–12)	0.92
**60**	32 (25–46)	29 (20–49)	0.35	28 (23–34)	24 (13–39)	0.60
**0.15**						
**20**	17 (14–27)	17 (13–24)	0.60	7 (5–11)	7 (5–8)	0.25
**40**	28 (19–38)	23 (16–34)	0.17	15 (14–17)	15 (11–18)	0.60
**60**	35 (21–44)	39 (23–49)	0.12	28 (20–41)	25 (16–37)	0.75
**0.3**						
**20**	16 (14–17)	17 (13–25)	0.69	8 (3–12)	7 (2–11)	0.12
**40**	26 (19–48)	25 (18–35)	0.25	14 (12–29)	13 (8–21)	< 0.05
**60**	38 (37–58)	35 (22–62)	0.46	30 (24–51)	22 (15–39)	< 0.05
**0.6**						
**20**	14 (5–16)	13 (9–16)	0.92	29 (18–35)	25 (18–31)	0.08
**40**	19 (10–23)	17 (12–21)	0.60	31 (19–52)	27 (16–43)	< 0.05
**60**	23 (14–26)	21 (16–26)	0.92	41 (28–57)	33 (22–44)	< 0.05
**0.9**						
**20**	26 (18–29)	27 (22–31)	< 0.05	23 (18–29)	20 (14–29)	<0.05
**40**	34 (30–42)	36 (27–42)	< 0.05	44 (31–47)	39 (25–42)	< 0.05
**60**	38 (30–42)	42 (33–51)	< 0.05	50 (43–60)	43 (39–45)	< 0.05
**1.2**						
**20**	26 (22–29)	29 (26–34)	< 0.05	17 (14–23)	9 (8–10)	< 0.05
**40**	32 (28–33)	35 (30–38)	< 0.05	36 (26–38)	21 (19–22)	< 0.05
**60**	32 (30–37)	41 (37–44)	< 0.05	42 (36–48)	32 (25–35)	< 0.05

Clot lysis (%) of clots exposed to streptokinase concomitantly with continuous-wave ultrasound at different intensities (n = 6 for each intensity) for one hour (US+SK) and clots exposed to streptokinase alone (n = 6 for each intensity, (control clots)) and in clots exposed to reteplase concomitantly with continuous-wave ultrasound at different intensities (n = 6 for each intensity) for one hour (US-r-PA) and reteplase alone (n = 6 for each intensity, (control clots)). Results are presented as medians and 5^th ^– 95^th ^percentiles. Wilcoxon's signed rank test was used to assess statistical difference.

### Reteplase treated clots

In the experiments using r-PA, statistically significant increases in clot lysis were seen following CW-US exposure at intensities of 0.05 W/cm^2 ^(3%, P < 0.05), and at 0.3 W/cm^2 ^following 40 and 60 min of exposure (1%, P < 0.05 and 8%, P < 0.05) and at 0.6 W/cm^2 ^following 40 and 60 min (5%, P = 0.03 and 8%, P < 0.05) of exposure. Increased clot lysis was seen at all times following CW-US exposure at intensities of 0.9 and 1.2 W/cm^2 ^(0.9 W/cm^2^: 20 min: 3%, P < 0.05, 40 min: 4%, P < 0.05, 60 min: 7%, P < 0.05 and at 1.2 W/cm^2^: 20 min: 8%, P < 0.05, 40 min: 15%, P < 0.05, 60 min: 10%, P < 0.05). No significant decrease in lysis was seen at any time or US intensity in clots treated with r-PA (see Table 1 and Figure 2).

## Discussion

The use of high frequency US to enhance thrombolysis during the treatment of stroke has shown promising results [1,3]. However, clot lysis levels *in vivo *have not been in the same levels as those reported *in vitro *[7,9,10]. This may well be due to the attenuation of intensity as US passes through the temporal bone during high-frequency US exposure [11], although direct comparison between *in vitro *and *in vivo *results should be made carefully. In the CLOTBUST trial, an intensity of 0.75 W/cm^2 ^was used [1], which would result in intensities between 0.01 and 0.06 W/cm^2 ^(following attenuation) reaching the obstructed vessel and the thrombus. We previously observed no enhanced fibrinolytic effects during pulsed-wave US exposure of r-PA-treated clots in this range of intensities [18], however effects were seen in the small intensity range between 0.125 and 0.25 W/cm^2^. In the present study, using CW-US exposure, a statistically significant increase in lysis of r-PA treated clots was seen at low intensity (0.05 W/cm^2^, 3% increase, P = < 0.05). Thus, applying high-frequency CW-US to r-PA treated stroke patients may improve clinical results. However, different frequencies were used in the present study (1 MHz) and in earlier clinical studies (2 MHz) [1,3], and the results should therefore be compared with care, also direct comparison between in-vitro and in-vivo results should be made with care. Another explanation could be that the number of patients included in the CLOTBUST-trial was to small to achieve statistical significance, this despite efforts to include a sufficient number of patients [24].

In the present study, increasing enhancement of the clot lysis was seen in the experiments on r-PA treated clots (intensities ≥ 0.3 W/cm^2^), which are intensities higher than can be expected after passing through the skull bone [11]. In the present experiments on r-PA treated clots, enhancement of lysis was at lower intensities compared to our earlier study using pulsed-wave US exposure [18]. However, it would be difficult reach such levels of intensity both with pulsed and CW-US, due to the high attenuation of the skull bone and considering the limited levels of out put intensity recommended in transcranial Doppler US [25].

In the present study no enhanced effects of SK were seen at any intensity level used. This is a contradictory result when compared to results from studies with pulsed-wave US exposure [16,17]. Thus, the mechanisms by which US enhance clot lysis might vary between CW-US exposure and pulsed-wave US exposure. This study shows decreased effects of clot lysis at intensities ≥ 0.9 W/cm^2^, a finding that has been seen earlier during pulsed-wave US exposure [16,17], however at higher intensities (≥ 2 W/cm^2^). This might indicate that also duty cycle is an important factor influencing the results in US-enhanced clot lysis, not only intensity. Streptokinase is still considered by some to be a useful thrombolytic drug in the clinical setting [26,27]. However, it does not appear to be suitable, in the setting of US enhanced thrombolysis, based on the decrease in effects induced by US exposure, according to the results in the present and earlier studies [16,17]. In the earlier studies it was possible to modulate the stereochemistry of SK by exposing it to US of different intensity levels of pulsed US. These effects occurred at US intensities below the prescribed upper limit of exposure of the human body to US energy (Mechanical Index < 1.9) [17]. Therefore, during SK treatment of any thrombotic disorders, possible undesired effects of exposure to US should be considered. And when using US to enhance the effects of SK in clinical situations, we recommend it to be restricted since reliable calculations and measurements of local US intensities in the treatment area is hard to perform. Streptokinase has also been shown to be associated with a higher risk of intracranial bleeding than other thrombolytic drugs, and is therefore not recommended for clinical use in the treatment for stroke [28].

We have previously demonstrated a direct effect on the thrombolytic substance during exposure with pulsed-wave US exposure, effects associated with both decreased and increased effects on clot lysis [17,18]. This was not examined in the present study, and we therefore do not know whether this effect exist when using CW-US. A recent study failed to reveal any changes in enzymatic activity of both SK and r-PA following US exposure [29]. Direct effects on the molecules of thrombolytic drug following CW-US exposure must therefore be investigated in the future.

### Limitations

The use of pure 0.9% NaCl solution as medium for experiments of fibrinolysis might not be the optimal solution for exploring when fibrinolytic effects are optimised. Previous studies have explored fibrinolytic effects in pure 0.9% NaCl solution showing them to be stable or partly reduced, but not totally inactivated [30-32]. Results from earlier studies [16,33] in vitro adding fibrinolytic drugs to pure 0.9% NaCl solution have been reproduced and verified in vivo [34,35] as well as in clinical studies [36]. The use of pure 0.9% NaCl solution without addition of plasminogen might explain the limited levels of clot lysis seen in the present study [37,38]. How this affects the results in the present method has to be studied in the future.

## Conclusion

Increasing intensities of CW-US exposure resulted in increased clot lysis of r-PA-treated blood clots, but decreased clot lysis of SK-treated clots. Continuous-wave US may thus be useful in US-enhanced clot lysis during stroke treatment with r-PA.

## Competing interests

The authors declare that they have no competing interests.

## Authors' contributions

BMH designed the investigation, performed the experiments, the statistical analysis and interpretation of the results, as well as the preparation of the manuscript. JC assisted with the statistical analysis and the preparation of the manuscript. AR supervised and designed the investigation as well as participated in the preparation of the manuscript. All authors read and approved the final manuscript.

## Pre-publication history

The pre-publication history for this paper can be accessed here:


